# Development and optimisation of a multi-component workplace intervention to increase cycling for the Cycle Nation Project

**DOI:** 10.3389/fspor.2022.857554

**Published:** 2022-10-26

**Authors:** Hayley Connell, Greig Logan, Camilla Somers, Graham Baker, Sarah Broadfield, Christopher Bunn, Luke D. Harper, Paul Kelly, Emma McIntosh, Jill P. Pell, Jill Puttnam, Sam Robson, Jason M. R. Gill, Cindy M. Gray

**Affiliations:** ^1^Institute of Health and Wellbeing, University of Glasgow, Glasgow, United Kingdom; ^2^School of Cardiovascular and Metabolic Health, University of Glasgow, Glasgow, United Kingdom; ^3^Physical Activity for Health Research Centre, University of Edinburgh, Edinburgh, United Kingdom; ^4^British Cycling, Manchester, United Kingdom; ^5^HSBC UK, London, United Kingdom

**Keywords:** cycling, workplace intervention, active travel, intervention development, co-design, evaluation

## Abstract

The Cycle Nation Project (CNP) aimed to develop, test the feasibility of and optimize a multi-component individual-/social-level workplace-based intervention to increase cycling among office staff at a multinational bank (HSBC UK). To do this, we first explored barriers to cycling in a nationally-representative survey of UK adults, then undertook focus groups with bank employees to understand any context-specific barriers and ways in which these might be overcome. These activities led to identification of 10 individual-level, two social-level, and five organizational-level modifiable factors, which were mapped to candidate intervention components previously identified in a scoping review of cycling initiatives. Interviews with HSBC UK managers then explored the practicality of implementing the candidate intervention components in bank offices. The resultant pilot CNP intervention included 32 core components across six intervention functions (education, persuasion, incentivisation, training, environmental restructuring, enablement). Participants received a loan bike for 12-weeks (or their own bike serviced), and a 9-week cycle training course (condensed to 6 weeks for those already confident in basic cycling skills), including interactive information sharing activities, behavior change techniques (e.g., weekly goal setting), bike maintenance training, practical off-road cycling skill games and on-road group rides. Sessions were delivered by trained bank staff members who were experienced cyclists. The CNP pilot intervention was delivered across three sites with 68 participants. It was completed in two sites (the third site was stopped due to COVID-19) and was feasible and acceptable to both women and men and across different ethnicities. In addition, the CNP intervention was successful (at least in the short term) in increasing cycling by 3 rides/week on average, and improving perceptions of safety, vitality, confidence, and motivation to cycle. Following minor modifications, the long-term effectiveness and cost-effectiveness of the CNP intervention should be tested in a full-scale randomized controlled trial.

## Introduction

Cycling is associated with several physical and mental health benefits, including lower risk of all-cause mortality (1–3), lower incidence of cardiovascular disease (3, 4), type 2 diabetes (5), stroke (6), hypertension (7), and breast and colon cancer (8), as well as, increased cardiorespiratory fitness (9), improved body composition (9), lower levels of stress, anxiety and depression (10), and improved wellbeing (11, 12) and cognitive function (13, 14). The largest health gains occur in the transition between not cycling, or cycling infrequently, to more regular cycling, rather than from regular cyclists further increasing their cycling volume (15). In addition, if cycling replaces motorized transport, it can reduce air pollution (16), carbon emissions (15) and congestion (17). The UK has low levels of cycling compared with several other European countries (18, 19) with only 11% of adults in England reporting cycling at least once a week (20). Nevertheless, there appears to be a latent demand for cycling, with surveys reporting that over half of UK adults would like to cycle more (21). Thus, with appropriate strategies, the potential to increase cycling in the UK, and to realize the multiple individual and public health, economic and societal benefits associated with this, is substantial.

The socio-ecological model suggests that interventions to promote cycling can be targeted at multiple levels including: (i) individual, (ii) social (including organizational), and (iii) built-environment (22, 23). A considerable body of evidence exists for the effectiveness of built-environment approaches to increasing cycling, but fewer studies have evaluated individual- and social-level behavioral initiatives, with mixed effectiveness to date (24). However, research in European countries with good cycling infrastructure has demonstrated that focusing solely on improving the built environment is not sufficient to maximize cycling participation (21, 25). Therefore, increasing cycling—which is a multifaceted behavior performed across multiple domains (commuting, utility, and leisure)—requires the range of barriers that prevent people from taking up and sustaining cycling to be addressed (26). At an individual level, barriers include lack of cycling skills and confidence (27, 28), feelings of physical discomfort (29), perceptions of effort (30) and lack of safety (31), and cost (32). At a social level, family, friends and work colleagues have also been shown to influence people's attitudes toward cycling (33).

The workplace is increasingly recognized as an important setting for delivery of health promotion interventions (34). Workplaces have the potential to encourage healthy behaviors—such as utility and leisure, as well as commuting, cycling—through adopting appropriate policies and promoting a supportive culture (35). In addition to being beneficial for the employee, workplace-based cycling interventions may provide benefits to the employer, including increased productivity (36) and reduced absenteeism (37). Whilst a number of workplace cycling initiatives have been trialed, many have focused on single components, such as cycle reward schemes (37), salary-sacrifice cycle purchase schemes (38), cycle challenges (39), or one-off cycle events (40). Few workplace initiatives have adopted an integrative approach, targeting both individual and social barriers to cycling over a consolidated period of time, which is likely to be necessary to maximize effectiveness given that most people report multiple barriers acting at different levels (41).

The aim of the Cycle Nation Project (CNP) was therefore to develop and pilot a multi-component individual-/social-level workplace-based intervention to increase cycling amongst people who cycle infrequently or not at all. In this paper we report the conceptualization and co-design of the CNP pilot intervention (Phase 1), and subsequent feasibility testing and optimization (Phase 2) using the 6SQuiD model (42) as a framework for intervention development.

## Phase 1—Conceptualization and co-design

The CNP was set up as a collaboration between academics at the Universities of Glasgow and Edinburgh, and key stakeholders [British Cycling (a UK cycling governing body), and the multinational bank, HSBC UK] to increase cycling across the UK. The approach aimed to develop an intervention to increase cycling participation amongst staff at HSBC UK offices, which, if successful, could be rolled out more widely across other similar organizations nationally and internationally.

Phase 1 encompassed the first five steps of the 6SQuID model (42), as shown in Table 1. Initially, to build on existing evidence, further define the causes of problem (low levels of cycling) and identify which of these might be changed in our target population (6SQuID steps 1 and 2), we performed a secondary analysis of data on barriers and attitudes to cycling_from a nationally-representative, cross-sectional survey of UK adults. We then conducted focus groups with HSBC UK employees to: first, understand the relative importance to our target population of the barriers identified by the UK-wide survey; second, explore any additional context-specific barriers; and third, identify ways in which the barriers might be overcome. The findings were mapped onto the results of an earlier CNP systematic scoping review of group and organizational initiatives to promote cycling (24) to identify candidate intervention components and the mechanisms of change (6SQuID Step 3). Finally, individual face-to-face interviews with HSBC UK office managers and co-design workshops with HSBC UK staff were used to finalize the pilot CNP intervention core components, Program Theory and delivery format (6SQuID Steps 3-5) for the Phase 2 feasibility study (6SQuID Step 6).

**Table 1:** An overview of the methods used to address the six steps in quality intervention development (6SQuID) in the Cycle Nation Project.

**6SQuID step**	**Methods**
1. Define and understand the problem and its causes.	• Secondary analysis of national survey data •Six focus groups with HSBC UK employees
2. Clarify which causal or contextual factors are malleable and have greatest scope for change.	• Six focus groups with HSBC UK employees (as above)
3. Identify how to bring about change: the change mechanism.	• Mapping exercise •Face-to-face interviews with five HSBC UK office managers •Co-design workshops with 10 HSBC UK staff
4. Identify how to deliver the change mechanism.	• Face-to-face interviews with five HSBC UK office managers (as above) •Co-design workshops with 10 HSBC UK staff (as above)
5. Test and refine on small scale.	• Face-to-face interviews with five HSBC UK office managers (as above) •Co-design workshops with 10 HSBC UK staff (as above) •Phase 2 feasibility study
6. Collect sufficient evidence of effectiveness to justify rigorous evaluation/implementation.	• Phase 2 feasibility study (as above)

Ethical approval for Phase 1 data collection was obtained from the University of Glasgow College of Social Sciences Ethics Committee (Ref. 400170195). All participants provided written informed consent.

### Defining the causes of the problem and scope for change

#### Secondary analysis of nationally-representative survey

In 2017, British Cycling commissioned a nationally-representative UK-wide survey of barriers and attitudes to cycling. The survey involved 5,000 respondents at four, quarterly timepoints between May 2017 and March 2018 (total *N* = 20,000) to account for potential seasonal variation in cycling. We conducted a descriptive secondary analysis of the survey data that focused on responses to two questions. The first, “What currently stops you from cycling or cycling more often?”, was asked of a subset of 14,999 respondents who reported not cycling as much as they could. The second, “Why do you have no interest in cycling?”, was asked of a subgroup of 2,347 respondents who reported they were not interested in either starting cycling or cycling more often. Respondents were stratified into: “Never Cyclists” (those who had never cycled or not cycled since childhood); “Lapsed Cyclists” (cycled in adulthood but not in the past year); “Occasional Cyclists” (cycled in the past year, but less frequently than once per month); “Regular Cyclists” (cycled between once per month and once per week); and “Frequent Cyclists” (cycled more than once per week).

The results are summarized in Table 2. We focused on responses from Never, Lapsed and Occasional Cyclists as representative of the CNP intervention target population (people who cycle infrequently or not at all). Amongst these groups, lack of perceived safety, lack of confidence riding on the roads and bad weather were the most highly cited barriers, reported by about half of respondents. Lack of time, particularly with respect to home, family and work commitments, was also a common barrier, particularly amongst Occasional Cyclists. Lack of knowledge of local places to cycle and unsuitable terrain were reported as barriers by about a quarter of respondents. Other barriers were less frequently mentioned, and interestingly, relatively few respondents reported cost as being important. Amongst the subgroup of respondents who did not want either to start cycling or to cycle more, the most frequently reported reasons were not liking cycling as a sport or preferring other sports, highlighting a perception of cycling as a sport rather than as an everyday activity. Concerns about the safety of cycling were also reported by this group.

**Table 2:** Responses to questions about barriers to cycling in a nationally-representative UK survey.

**Question**	**What currently stops you from cycling or cycling more often?** **(*****n*** = **14,999 respondents reporting that they did not cycle as much as they possibly could)**				
	**Cycling category**				
	**Never cyclists** ** (*n* = 5,439)**	**Lapsed cyclists** ** (*n* = 5,128)**	**Occasional cyclists** ** (*n* = 2,766)**	**Regular cyclists** ** (*n* = 914)**	**Frequent cyclists** ** (*n* = 842)**
The cycling (on-road) infrastructure does not make me feel safe	52.8%	55.3%	45.6%	42.6%	34.9%
I don't feel confident riding my bike on roads	54.4%	54.2%	43.4%	35.5%	29.2%
Bad weather puts me off cycling as a hobby	46.2%	47.0%	51.7%	46.0%	35.3%
I do not have the time owing to home/family commitments	26.7%	28.4%	43.6%	39.5%	32.9%
I do not have the time owing to work commitments	21.3%	22.9%	41.9%	37.8%	33.9%
I don't know of any facilities where I can cycle	30.7%	27.4%	25.0%	21.9%	18.7%
The local terrain doesn't suit me	29.1%	28.0%	23.5%	19.9%	15.7%
Cycling requires too much faffing around	30.9%	21.1%	19.9%	14.0%	16.6%
I do not have the time owing to other leisure/social commitments	18.4%	19.4%	30.4%	25.7%	24.9%
Cycling is too expensive	23.8%	17.2%	14.3%	14.4%	14.4%
There are not enough cycling events in my area	17.2%	13.8%	18.4%	18.6%	17.9%
I am not interested in sport	41.4%	25.7%	21.1%	23.3%	17.9%
Cycling is not safe	22.2%	22.0%	14.5%	0.0%	6.0%
I prefer other sports	16.0%	22.7%	25.3%	14.0%	16.4%
Cyclists do not behave safely	15.6%	15.2%	7.2%	2.3%	3.0%
Cycling is too expensive	6.9%	5.9%	3.6%	2.3%	1.5%
I don't like cycling clothes	6.7%	5.6%	6.0%	16.3%	3.0%
There is too much doping in professional cycling	3.3%	3.7%	0.6%	7.0%	1.5%
Cycling excludes women	0.2%	0.5%	0.6%	0.0%	0.0%
I cycle already but I have no desire to cycle more often	0.1%	2.2%	29.5%	60.5%	61.2%
Other reason	19.1%	29.4%	5.4%	7.0%	9.0%
Don't know	10.8%	12.3%	18.1%	4.7%	11.9%

Percentages refer to those agreeing or agreeing strongly to the question on a five-point scale.

#### Focus groups

Focus groups were then conducted at six HSBC UK workplaces (Manchester, London, York, Birmingham, Liverpool and Edinburgh) between August and November 2018. The sites were selected to represent geographical diversity across the UK. Staff were sent an internal email asking those who had cycled less than once a month in the past year if they were interested in taking part. The focus groups (each 6–8 participants, mean *N* = 7) were conducted by HC, an experienced qualitative researcher, and lasted on average 77 min (range 63–90 min).

The discussions were digitally recorded and transcribed verbatim with participant consent. Anonymized transcripts were analyzed using a thematic framework approach (43) and NVivo12 software to organize the data. Two transcripts were read independently by three members of the research team with expertise in qualitative methods (HC, GL, CMG), who then met to agree a coding frame. The coding frame was applied to all transcripts by HC and GL, and 13 broad themes; “Time,” “Safety,” “Environment,” “Effort,” “Views toward bikes/e-bikes,” “Other people,” “Storage,” “Security,” “Infrastructure/facilities,” “Education/training,” “Cycle schemes,” “Maintenance,” and “Incentives” were identified. These were then explored in detail to identify 10 broad barriers and 22 associated specific factors limiting cycling among HSBC UK staff, displayed in Table 3. Similar to the survey, lack of time due to competing family and work commitments, and safety concerns emerged as important barriers (for women in particular); the latter stemmed from a lack of confidence about cycling on roads (including cycling in traffic) and perceptions of poor cycling infrastructure. Furthermore, in relation to effort, unsuitable terrain (i.e., hills and distance) was reported as discouraging people from cycling. The cost of cycling (not just the bike itself, but also the cost of associated equipment) appeared to be more important for focus group participants than survey respondents. Some also complained about lack of social support (including support/guidance from more experienced cyclists) and not having anywhere to store a bike at home (lack of space and/or living in a flat). Others described how a lack of workplace facilities (showers, lockers, bike storage) and not having a supportive workplace culture for cycling (including not being able to wear appropriate clothes for cycling) deterred them from commuting by bike. Some also felt they lacked the skills and experience needed to cycle safely from place to place and to maintain their bike in a road-worthy condition. Finally, despite HSBC UK participating in a subsidized bike purchase cycle-to-work scheme, many were unclear how to use this. They also reported finding local cycle-share schemes, designed to help people access affordable cycling, difficult to navigate.

**Table 3:** Broad barriers and specific factors limiting cycling identified by HSBC UK staff focus group participants.

**Broad barrier**	**Specific factor**	**Supporting extract**
Lack of time	Family and/or work commitments	*I'd like to do more, but for me it's just lack of time. I don't get a minute to myself of a morning or of a night really. I couldn't structure it in… (Participant 4, M, Liverpool)*
Lack of perceived safety	Lack of confidence	*At the moment, I'm afraid on my bike. I'm not completely confident of the route in and out to work (Participant 2, F, Edinburgh)* …*we don't know how to deal with icy footpaths, we don't know how to deal with crosswinds, we don't know how to deal with, you know. So, it's the actual physicality of being safe (Participant 2, F, Edinburgh)*
	Poor environmental infrastructure	*We need to improve the infrastructure more than anything else…you'll get cycle paths and then all of a sudden, no cycle path. And you're just stuck in the middle of the road (Participant 3, M, Manchester)*
	Poor road surfaces	*[the] bike riding side just reminds me of potholes…I just don't think our roads are great (Participant 5, M, Liverpool)*
	Traffic	…*I'm afraid to stay on the road, because I'm afraid of the cars, the traffic, or I don't have confidence with traffic (Participant 2, F, Edinburgh)*
Effort	Hills	*It's quite a hilly country….I don't want to cycle up a hill. I want to cycle on a flat, serene ground (Participant 5, M, Manchester)*
	Distance	…*if I worked a lot closer to home, that would assist, cos then I wouldn't think about the taking the car. Even if I worked, like, five or six miles away, I could bike. But that's unlikely to be able to happen (Participant 2, F, York)*
Cost	Cost of bike	“*I've been talking about, with my wife, that we should get a bike, but they're quite expensive and, you know, you just…you know, kind of, postpone it and postpone it (Participant 6, M, Manchester)*
	Cost of equipment (e.g., helmet and clothing)	*There's quite a high barrier to entry if you want to buy a bike and not stand out like a sore thumb…because you spend at least a few hundred quid on a road bike and then you look at clips, shoes, lycra, gloves, helmet, jersey, two or three water bottles…It's a grand before… (Participant 3, M, Birmingham)*
Lack of social support	Work colleagues, friends, family not interested in cycling	*Me and my husband have got a bike and I've asked him loads of times, but he's just not bothered (Participant 2, F, Birmingham)* *For me I mainly cycle at home, and the limiter for me would be friends, because none of my friends really do it (Participant 2, M, London)*
	No cycling role model/mentor	…*if I had a buddy, that would teach me the etiquette, what to do when I'm coming across someone, you know…who's got right of way (Participant 2, F, Edinburgh)*
Lack of facilities at home	Practical and secure storage	*I live in a very small flat…I've got nowhere to keep a bike (Participant 3, F, London)* *I'd have no space to put the bike away. Just…prams, there's kids' toys…the house is just one big nursery basically. I'd have no space (Participant 4, M, Liverpool)*
Lack of facilities at work	Secure storage	*If you're going to encourage everyone to bike to work, say 20/25 people are all coming here on a bike, then there's nowhere near enough places to leave your bike (Participant 5, F, York)*
	Showers	*More facilities at work like showers. At the moment we've got two showers, one in the men's, one in the ladies'. And I think…they're not private (Participant 5, F, York)*
	Lockers	…*there's a massive waiting list for a locker (Participant 5, M, London)*
Lack of cycle friendly workplace culture	Professional dress code	*It would be odd to just have some people who dress down…the whole culture would have to change (Participant 7, F, London)* …*and then I need to put my shirt in my bag and then that's going to be creased (Participant 1, M, York)*
	Bike spares/tools (inner tube, pump etc.)	*That sounds good, actually…like a maintenance station that's at your workplace. So if there is anything that's to happen, to go wrong, then you know you could fix it, or there's spare whatever, in the building (Participant 1, F, Edinburgh)*
Lack of skills	Unsure how to cycle on the road	….*I knew how to ride a bike, but I didn't know how to do it safely on the roads…I'm used to getting to places by trains or buses or Ubers, but I've never actively gone out to gain that education on how to ride bikes on the roads. It's mainly the safety element for me (Participant 1, F, Birmingham)*
	Lack of experience	…*some of these fears that we have it's probably because we never rode bikes on these busy* roads when we were kids. *(Participant 5, M, London)*
	Lack of bike maintenance skills	…*changing an inner tube, etc…is something people would need to know (Participant 5, M, Liverpool)*
Lack of knowledge	Unsure which cycle routes are suitable/how to plan a route	*I'm in the center of the city, so how do I get from point A to point B, to a point of safety (Participant 3, F, Edinburgh)*
	Lack of knowledge about cycle schemes (e.g., cycle-to-work)	*…the cycle to work scheme. That's quite good, isn't it. Do HSBC do that? (Participant 4, M, York)*

**Figure 1:**
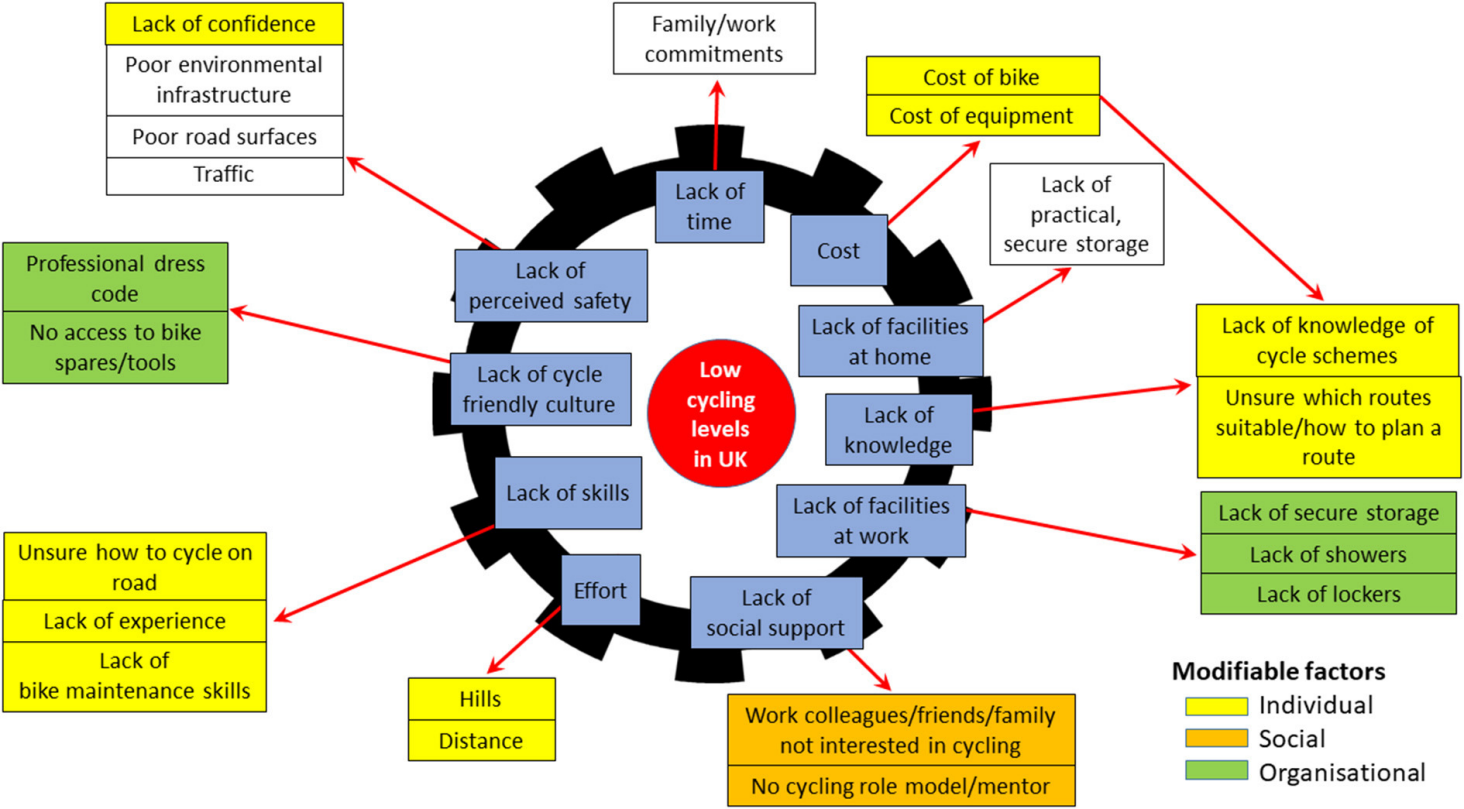
Barriers to cycling (shown in blue) and associated modifiable factors at individual, social and organizational levels.

Following discussion of the focus group findings, the CNP team (including the University researchers and British Cycling representatives) agreed on 17 (from the 22) specific factors that could potentially be modified within a group-based workplace intervention. These included 10 individual-level factors relating to lack of confidence, knowledge and skills, cost and effort (where it was agreed that e-bikes might overcome some of the issues related to hills and distance). Two social-level factors (namely having peers and mentors/role models to cycle with) were also addressable through a group-based intervention in the workplace. Finally, an additional five factors (professional dress code, access to spares/tools, and lack of bike storage, showers and lockers) were identified as being modifiable at the organizational level. A diagrammatic summary of all modifiable factors is provided in Figure 1.

### Identifying the change mechanism

A full day workshop involving five university researchers and two British Cycling representatives was held to map the activities (action types) reported by the earlier CNP systematic scoping review (24) and focus group participants' suggestions of how to support people to cycle more (facilitators) against the modifiable factors. The results of this theoretical mapping exercise are provided in Supplementary Table 1. It identified 68 candidate intervention components relating to 26 action categories (groups of components sharing a similar function) across seven intervention functions (IF) from Michie et al.'s Behavior Change Wheel (BCW) (44). The action categories included: increasing knowledge/understanding of the benefits of cycling, cycling safety, route planning and how to cycle, and signposting to cycling organizations (IF—Education); individual and group counseling and travel diaries (IF—Persuasion); material (e.g., loan of bike) and financial (e.g., work cycle scheme, free bike servicing, and safety equipment) incentivisation, and gamification/challenges (e.g., goal setting and certificates) (IF—Incentivisation); training courses (IF—Training); secure bike storage and changing/maintenance facilities, cycling personnel, mass participation events, group cycling, workplace policies (including flexible dress code and working hours) (IF—Environmental restructuring); buddying (IF—Modeling); provision of bikes/ebikes, accessories and maintenance, and signposting to cycle share schemes (IF—Enablement).

### Refining the pilot CNP intervention core components and delivery format

Telephone interviews with managers (all men) from five of the HSBC UK workplaces involved in the staff focus groups (Manchester, London, York, Birmingham, and Liverpool—no managers from Edinburgh were available for interview) then explored the practicality of implementing the CNP candidate intervention components within bank offices. The five interviews (mean duration 44 min, range 24–86 min) were conducted in January 2019 by GL and HC and audio recorded with participant consent. GL and HC listened to the audio recordings and took notes (including supporting extracts) to summarize the managers' views.

All managers were confident there were suitable outdoor facilities at their offices for delivering practical components of the CNP intervention:

*There's certainly space, absolutely, it's a huge branch with space to do that. I guess cost would come into it, but there is the facilities there, and they certainly could be extended*. Manager 2

They supported the idea of training staff volunteers who were enthusiastic cyclists to become Cycle Champions certified to deliver the CNP intervention. One noted that this sort of initiative aligned with current organizational policy within HSBC UK:

…*we've got some people who have done ride leader courses. We're encouraging more of our staff to do that*. Manager 1

There was broad agreement that provision of bikes (including the option of ebikes) would be essential to encourage participation in the intervention:

*I think the idea of a loan bike would be a really good idea and maybe a loan bike for a period of time of a week or a month or maybe longer. So, the people who haven't got those could give it a proper go…* Manager 3

However, there was little support for the introduction of a flexible dress-code. Managers felt this could be difficult to implement, particularly where staff were working in customer-facing roles:

*I guess my view is I would be concerned about any item of clothing that can go through the wear and tear of cycling through the elements and still look smart and professional in a banking environment, that would be the challenge*. Manager 2

Although the adoption of a flexible working hours policy was not immediately dismissed, some managers felt in smaller branches there might be issues around implementation that would require careful negotiation:

…*a lot of our branches are open at 8 a.m. and close at 6 p.m…. by arrangement… if one of my team said to me, I want to come in early and leave late… I want to cycle to work every day… I might be able to accommodate somewhere in-between, say for two days a week, you can do that…* Manager 4

**Figure 2:**
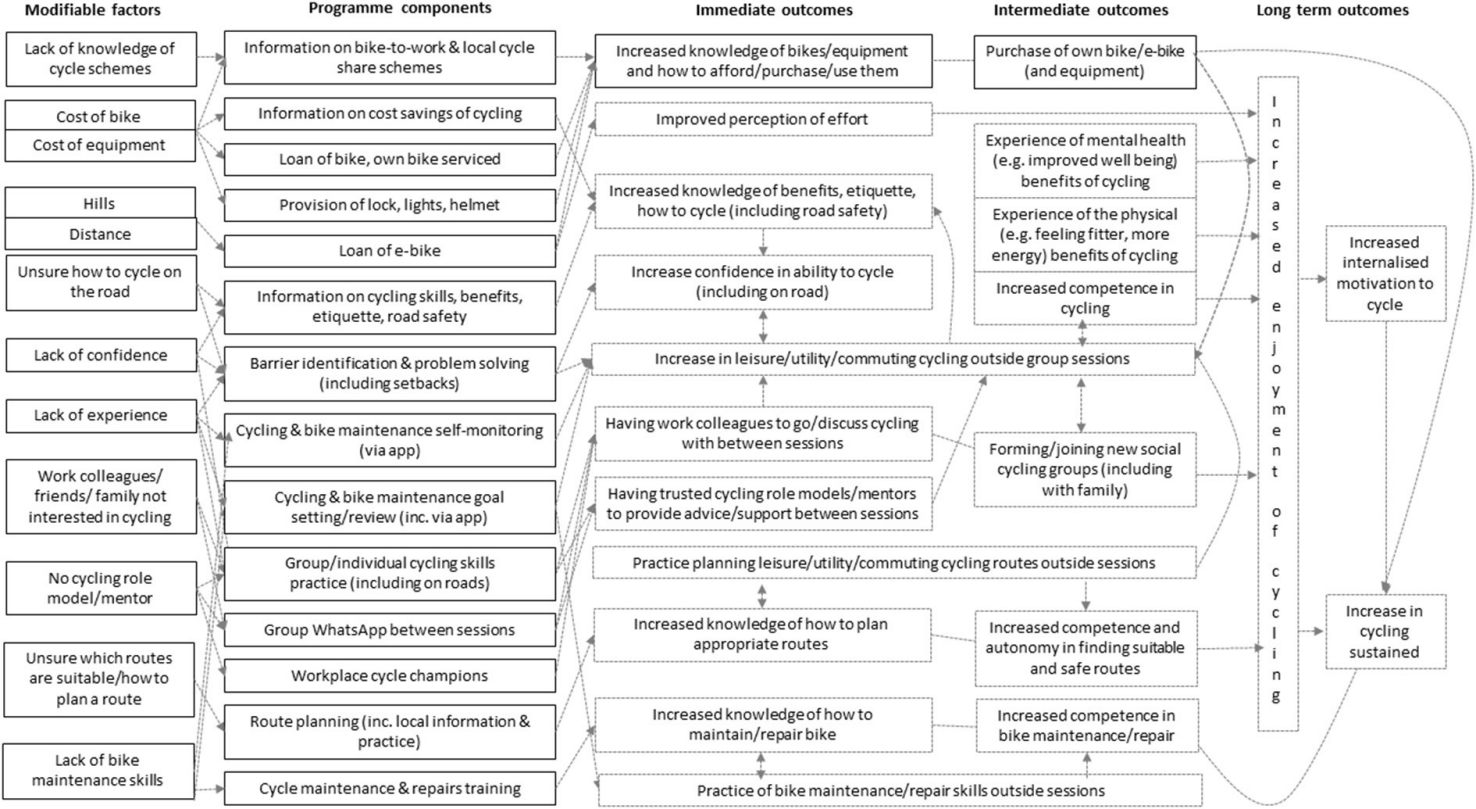
The Cycle Nation Project program theory.

The CNP research team (including representatives from British Cycling and HSBC UK) used the findings from the manager interviews to: first, confirm 32 core components relating to 16 action categories across six BCW intervention functions for inclusion in the pilot CNP intervention (summarized in Table 4); and second, finalize the CNP Program Theory. As Figure 2 shows, as well as drawing on the evidence from the theoretical mapping exercise, staff focus groups and manager interviews, the CNP Program Theory was also informed by Self-Determination Theory (45), which suggests that people are more likely to initiate and sustain a new behavior (such as cycling) if their motivation to perform a behavior is internally (rather than externally) regulated.

**Table 4:** Cycle Nation Program intervention core components identified following theoretical mapping and manager interviews.

**Intervention function**	**Action category – CNP core components**
**Education** Increasing knowledge or understanding	**Increasing knowledge or understanding of benefits of cycling**
	–*Information on time-saving benefits of cycling (SR)*
	–*Information on cost benefits of cycling (SR)*
	–*Information on time taken to cycle route (e.g., in app) (FG)*
	**Increasing knowledge or understanding of cycling safety**
	–*Information on cycling safely (SR, FG)*
	–*Information on cycle étiquette (FG)*
	–*Acting out travel scenarios (SR)*
	**Route planning/personal and individualized travel planning**
	–*Information on accessibility and local routes (SR, FG)*
	–*Travel and safe-route maps (SR, FG)*
	–*Digital cycling apps (SR, FG)*
	–*Cycling website (SR, FG)*
	**Practical or instrumental information**
	–*General practical “Everything you need to know about cycling” information (SR)*
	**Signposting to cycling resources/organizations**
	–*Cycling-related contacts (SR)*
**Persuasion** Communication to induce +ve/–ve feelings or stimulate action	**Group counseling**
	–*Group counseling (including barrier identification and problem solving) to increase cycling (SR)*
**Incentivisation** Creating expectation of award	**Material**
	–*Bikes for attending sessions (SR)*
	**Financial**
	–Subsidy, salary sacrifice, tax free loan for buying bike and equipment (SR, FG)*
	–*Cycling-related gifts*
	–*Free bike service for taking part*
	**Gamification/challenges**
	–*Goal setting and personal challenges (SR)*
	–*Awards and certificates (SR)*
	–*Active games (SR)*
**Training** Imparting skills	**Training courses and sessions**
	–*Cycle skills, proficiency and safety training and courses (SR, FG)*
	–*e-Bike skills and proficiency training and courses (SR, FG)*
	–*Maintenance skills courses (FG)*
	–*Independent skills practice (SR)*
**Environmental restructuring** Changing the social environment	**Group cycling**
	–*Led group bike rides (SR,FG)*
	**Workplace or organizational policies**
	–*Training internal staff to become certified cycling instructor (SR)*
**Enablement** Increasing means/reducing barriers to increase capability or opportunity	**Provision of bike accessories**
	–*Safety equipment (helmets, lights, reflective strips) (SR, FG)*
	**Provision of eBikes**
	–*Loan of eBike to use during intervention/eBike trial before purchase (SR, FG)*
	**Provision of bikes**
	–*Short term hire or lease of bike during intervention (SR, FG)*
	–*Information on how to access shared cycle schemes (FG)*
	**Provision of bike maintenance**
	–*General bike maintenance (SR, FG)*
	–*Bike repairs (SR, FG)*

SR, Scoping Review; FG, Focus Groups.

^*^Existing HSBC UK cycle-to-work scheme.

The core components and CNP Program Theory were then used to develop the delivery protocol and participant handbook for a practical 9-week cycling intervention and an associated 2-day Cycle Champion training course. These resources drew on existing British Cycling materials, where appropriate. A CNP app was also developed to support self-monitoring and goal setting for both cycling and bike maintenance.

In the final stage of intervention development, GL and HC conducted two co-design workshops with staff at HSBC UK offices in London (*N* = 5 staff) and York (*N* = 4 staff) to refine the intervention for delivery in the Phase 2 feasibility study. Detailed field notes were taken, and, together with audio-recordings of each workshop, were written up electronically to summarize participants' views.

One important concern raised during both workshops was that early off-road practical skills sessions might be too basic for some people. Some participants suggested that two levels of the intervention (one basic, one more advanced) might be needed to cater for different levels of confidence and experience:

*I think a one size fits all is not where you need to go with it… I don't need to see how to break, or turn a corner, I can do all of that stuff* . Workshop Participant 4, Female, York

*Maybe what you need is two different programmes, one for beginners…* Workshop Participant 5, Female, London

Overall, the core components were viewed favorably, and staff were particularly positive about the prospect of taking part in the CNP intervention alongside other co-workers with similar cycling skills and experience:

*The social content, being able to do it in a group, you're not alone… having the support of your peers, it's a strong incentive in itself*. Workshop Participant 4, Female, London

The final CNP intervention that was piloted in the Phase 2 feasibility study comprised two versions: a 9-week Foundation Course and a condensed 6-week Intermediate Course for participants who were already confident in basic cycling skills. The sessions combined interactive information sharing (including bike and personal safety, benefits of cycling, route planning), behavior change techniques (46) (including goal setting and review, self-monitoring of cycling, barrier identification and problem solving, social support, overcoming setbacks), bike maintenance training, and practical off-road cycling skill games and on-road group rides.

## Phase 2—Feasibility testing and optimization

In Phase 2, we conducted a mixed-methods before-and-after study to assess the feasibility (including recruitment, adherence and practical aspects of delivery), acceptability and potential effectiveness of the CNP pilot intervention [6SQuiD Step 6 (42)]. Three large HSBC UK offices were selected to take part in the feasibility study. These were chosen as having an adequate pool of potential participants, access to areas suitable for delivery of the off-road practical activities and adequate storage to allow participants to bring their bikes to work for intervention sessions, and to represent the geographical diversity of the UK, and The first delivery of the CNP pilot intervention took place at a central London office between August 6th and October 8th, 2019; the second at an office in a business park on the outskirts of Edinburgh between October 1st and November 27th, 2019; and the third at an office in a business park outside Southampton starting February 12th, 2020 (the COVID-19 pandemic halted this delivery in March 2020).

### Methods

#### Recruitment

We aimed to recruit 5–10 Cycle Champions and 20–30 participants at each office from staff members aged ≥18 years. Cycle Champions were recruited *via* an email from a central HSBC UK manager and were eligible if they were self-identified, competent cyclists. All underwent a 2-day CNP training course run at each of the three offices by a qualified British Cycling trainer and a University of Glasgow researcher (GL). The course was designed to be highly interactive and to provide experiential learning of how to deliver the CNP core components using the intervention delivery protocol, including: facilitating group discussions; encouraging the use of behavior change techniques; delivering practical training including bike maintenance and cycling skills; and leading group rides. Champions also underwent 1-day First Aid training certification.

Participants were recruited *via* email, office posters, face-to-face interactions with Cycle Champions (all sites) and an information session (London only). Staff were eligible if they were able to ride a bike but were currently cycling infrequently (less than once a month) or not at all. To confirm their eligibility, those who expressed interest in the study, were emailed a questionnaire asking, “Over the past 12 months, on how many occasions have you cycled?” and the Physical Activity Readiness Questionnaire [PAR-Q+ (47)] to confirm they did not have any contraindications to exercise. Once screened, participants were asked if they wanted to loan a bike for the duration of the intervention [and which type (e.g., ebike, hybrid, and folding) and model they would prefer] or have their own bike serviced.

#### Cycle nation project pilot intervention delivery

CNP sessions were delivered in the early evening immediately after work at London and Edinburgh sites, and during the lunch break at Southampton. The first three Foundation sessions and the first Intermediate session were delivered in traffic-free outdoor locations near each office. These were identified by Cycle Champions and included an unused basketball court near the London office and empty staff car parks in Edinburgh and Southampton. Once participants progressed to the on-road sessions, Cycle Champions identified low-traffic roads close to the offices for group rides. Although initially it was envisaged that the Foundation and Intermediate groups would meet separately, in practice these tended to be merged into a single session in later weeks.

Two large national bicycle shops with partnership agreements with British Cycling and HSBC UK were identified to supply loan bikes for 12 weeks (to cover the duration of the intervention and some additional weeks to allow participants to transition to buying their own bikes) or to provide servicing for those opting to use their own bike. Loan bikes were purchased by HSBC UK from the partnering bike shops for the Edinburgh and London deliveries and then pooled into a bike fleet for Southampton. Loan options included hybrid, road, folding and ebikes to suit a range of usage requirements. All participants also received helmets, locks and rear lights for taking part (participants at Edinburgh also received front lights to allow them to cycle safely during the late autumn/early winter evening sessions).

#### Data collection

To assess participant recruitment, the numbers of HSBC UK staff expressing an interest in CNP intervention, assessed for eligibility, and completing screening and baseline measures were recorded by the University of Glasgow research team. To assess intervention attendance and completion (adherence), Cycle Champions were asked to return session attendance registers *via* email to the University of Glasgow research team each week. In London, Cycle Champions asked any participants who missed sessions for their reasons for non-attendance, whereas in Edinburgh and Southampton, participants were telephoned by a researcher if absent from two consecutive sessions. Participants were judged to have completed the intervention if they attended at least two-thirds of available sessions. In-depth audio-recorded telephone interviews were conducted by HC with five of the 18 participants who did not complete the intervention in London and Edinburgh to explore reasons for non-attendance (mean duration 16 min; range 13–18 min).

To assess acceptability and the practical aspects of delivery, three face-to-face focus group discussions or paired interviews were held with participants who completed the intervention in London (one focus group with seven participants) and Edinburgh (one focus group with four participants and one paired interview) in the week after the intervention ended. Participants were invited by email to take part in focus groups/interviews held in their office during working hours. All focus groups/interviews were conducted by GL and lasted on average 58 min (range 50–64 min). Post-intervention telephone interviews with Cycle Champions were conducted by HC in London (*n* = 3) and Edinburgh (*n* = 5); they lasted on average 28 min (range 15–35 min). Finally, GL conducted three additional telephone interviews with Southampton participants to explore acceptability of the CNP app, which, due to delays during its development, only became available for the Southampton delivery (mean duration 10 min; range 5–14 min).

An interview schedule was used to guide focus group discussions/interviews around participants' views of the CNP pilot intervention, what was useful/not useful and what could be improved for future deliveries. Cycle Champions were also asked about the training, delivery materials and any adaptations they made during intervention delivery. The Southampton interviews focused on participants' views and experiences of using CNP app and its specific features, including self-monitoring and goal setting. All focus groups and interviews were audio recorded with participant consent.

To assess practical aspects of delivery and inform intervention optimization, selected delivery sessions (*N* = 25, including Foundation-only, Intermediate-only and Joint sessions) were observed across the three sites (London *n* = 10, Edinburgh *n* = 10, Southampton *n* = 5) by members of the research team (GL, HC, CMG). After each session, written summaries were completed electronically following an observation proforma focusing on how/if key components were delivered and any operational issues (e.g., timing, access to loaned bikes/servicing, and bike storage). Full details of which sessions were observed are provided in Supplementary Table 2.

To explore potential effectiveness, 1 week prior to starting and within 3 weeks of the end of the intervention, participants completed online questionnaires including self-reported frequency of total, leisure, commuting and utility (e.g., going to the shops) cycling. Other measures included perceptions of cycling and walking safety (48), motorized transport use, self-esteem [Rosenberg Self-Esteem Scale (49)], wellbeing [Warwick-Edinburgh Mental Wellbeing Scale, WEMWBS (50)], self-reported vitality [modified Subjective Vitality Scale, SVS (51)], and motivation for cycling [modified Behavioral Regulation in Exercise Questionnaire, BREQ-2 (52)].

At baseline, self-reported characteristics (age, gender, ethnicity, and bike ownership) and whether participants wanted to loan a bike/have their own bike serviced were also recorded, and researchers trained in standardized protocols visited each office to collect objective weight and height measurements. Weight (kg) was assessed using electronic scales (Tanita HD 352, Middlesex, UK) with participants removing shoes and emptying their pockets prior to measurement. Height (cm) was assessed using a portable stadiometer (Seca Leicester, Chino, CA, USA) with shoes removed. Each participant's body mass index (BMI) was calculated as weight (kg)/height (m)^2^. Finally, to ensure participant safety during the intervention, resting blood pressure was measured using a digital blood pressure monitor (Omron HEM-705CP, Milton Keynes, UK). Three participants with elevated blood pressure (systolic ≥140 mmHg and/or diastolic ≥90 mmHg) were encouraged to consult their GP before commencing the intervention, but were not excluded from taking part. At follow-up, participants were also asked to rate different aspects of the CNP pilot intervention, both specific content and overall, using a 5-point Likert scale where 1 = “Strongly Disagree” and 5 = “Strongly Agree”.

#### Analysis

Focus groups and interview audio recordings were transcribed verbatim. Anonymized transcripts were analyzed using a thematic framework approach (43) and NVivo12 software to organize the data. GL and HC read all transcripts to agree a coding framework comprising six themes based on the feasibility study research questions: i.e., feasibility, acceptability, potential effectiveness and optimization (a description of each theme is provided in Supplementary Table 3). They then applied the coding framework to all transcripts, double coding one interview and one focus group to ensure it was applied consistently. The framework was also applied to the electronic observation proformas by GL.

Recruitment, attendance and completion data were compiled in Excel spreadsheets to calculate summary descriptive data (numbers and percentages). We defined completion of the program as attendance of at least two-thirds of sessions. Questionnaire and measurement data were analyzed using SPSS version 22 (IBM Corporation, Armonk, NY). Participant baseline characteristics were reported using descriptive means and frequencies. Potential effectiveness was explored using paired *t*-tests to assess changes in outcomes between baseline and post-program. Significance was set at *p* ≤ 0.05.

#### Ethical approval

Ethical approval for Phase 2 was obtained from the University of Glasgow College of Medical, Veterinary and Life Sciences Ethics Committee (Ref. 200180138). All participants provided written informed consent.

### Results

#### Feasibility—Recruitment and adherence

**Figure 3:**
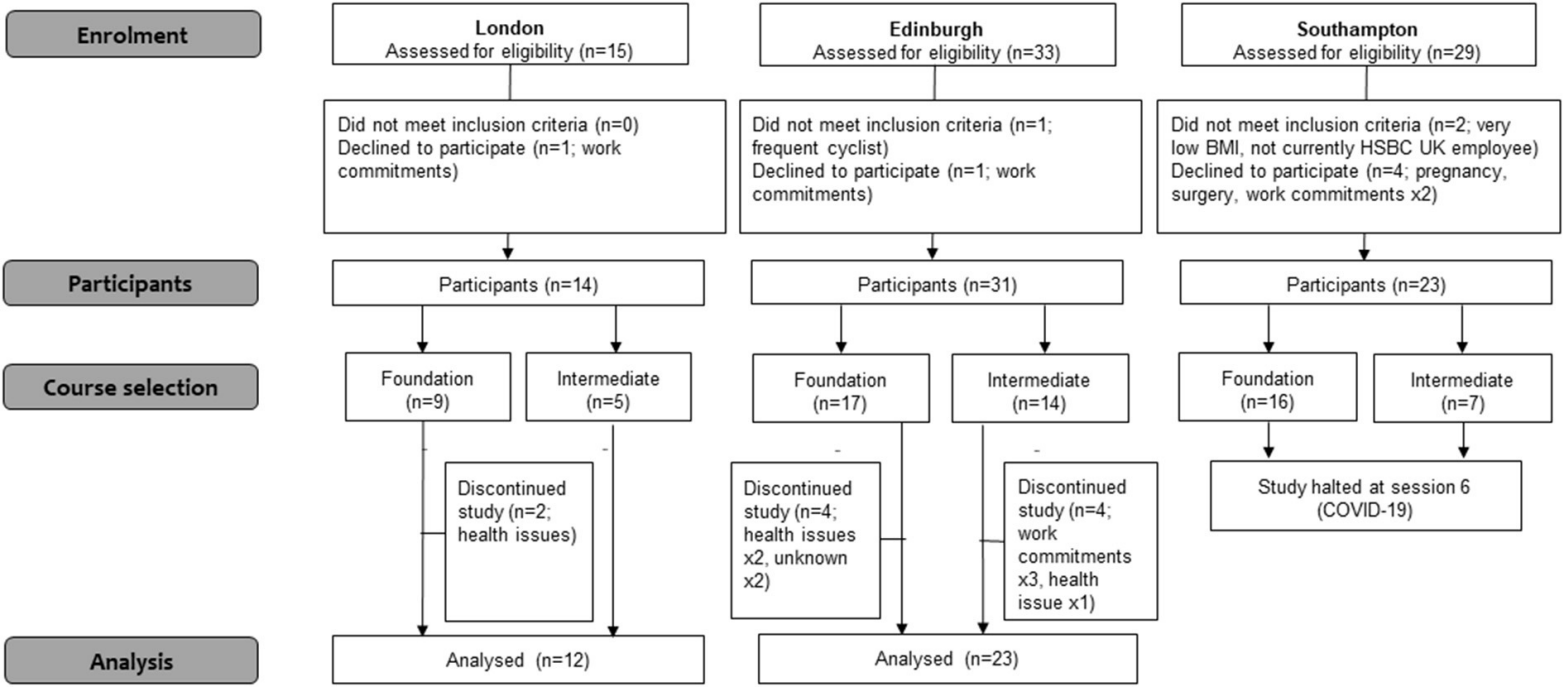
Flow of participants through the Cycle Nation Project feasibilty study.

Four Cycle Champions (all men) were recruited at the London office. Ten (8 men, 2 women) were recruited at the Edinburgh office, and eight (6 men, 2 women) were recruited at Southampton. Two Cycle Champions withdrew following training: one due to illness (London) and one due to issues with session delivery times (Edinburgh). As shown in Figure 3 and Table 5, a total of 68 HSBC UK office staff took part in the CNP pilot intervention across the three sites (London *n* = 14, Edinburgh *n* = 31, Southampton *n* = 23). Participants in were aged on average 39.8 (SD ± 10.0) years, and 54.4% were men. Over 60% were either overweight or obese, and over three quarters reported their ethnicity as white. More participants selected the Foundation course than the Intermediate course, and fewer than half already owned a bike.

**Table 5:** Participant baseline characteristics (*n* = 68).

**Participant characteristics**	**Mean ±SD or *n* (%)**
Age (years)	39.8 ± 10.0
**Gender (** ***n*** **, %)**
Male	37 (54.4)
Female	31 (45.6)
BMI (kg/m^2^)	27.1 ± 5.9
**BMI category (** ***n*** , %)*
Underweight	1 (1.5)
Normal	26 (38.2)
Overweight	23 (33.8)
Obese	18 (26.5)
**Ethnicity (** ***n*** **, %)**
White	52 (76.5)
Asian/British Asian	12 (17.6)
Other	4 (5.9)
**Course choice (** ***n*** **, %)**
Foundation	42 (61.8)
Intermediate	26 (38.2)
Bicycle ownership (*n*, %)	32 (47.1)

^*^BMI categories: underweight (<18.5 kg/m^2^), normal (≥18.5– <25 kg/m^2^), overweight (≥25– <30 kg/m^2^), obese (≥30 kg/m^2^).

Overall, participants attended 62.5% of sessions, and 60.0% (27/45) completed the intervention. London participants attended 61.5% of sessions, and 71.4% (10/14) completed the intervention (as defined by attending at least two thirds of available sessions). Attendance was similarly good in Edinburgh (63.4%) and extremely high in Southampton (98.1%) up to the point the program was suspended (after week 5) due to COVID-19. Full detail of attendance is provided in Supplementary Table 4. The clear structure of the program was valued by participants and motivated many to keep attending:

I knew what was coming up in every session, and that was helpful […] having a structured weekly detailed idea of what was going on that kept me going back. Foundation Participant 1, Female, Edinburgh

However, despite the good attendance in Edinburgh, completion (54.8%, 17/31) was lower than in London. Exit interviews suggested that this may have been due to winter weather and low light during evening sessions toward the end of the program. In addition, adherence tended to be poorer among Intermediate participants in Edinburgh, leading one local Cycle Champion to question whether running courses at two different levels was worthwhile (particularly given the logistical considerations of doing so):

The intermediate course to me pitched at the wrong level, it's too basic for…there's no need for it. Cycle Champion 3, Male, Edinburgh.

#### Feasibility—practical aspects of delivery

Of the 68 participants at baseline, 82.4% (56, London *n* = 10, Edinburgh *n* = 25, and Southampton *n* = 21) opted to loan a bike to take part in the intervention (this included some who already owned a bike). Over half of participants (53.6%) opted for hybrid bikes, 19.6% chose a road bike, 17.6% chose an e-bike and 8.9% selected a folding bike. Full detail of bike loans is provided in Supplementary Table 5.

It was originally envisaged that participants would store their bikes at home and bring them into work for program sessions. However, many participants left their loan bikes at their office for convenience:

…* there was a big faff ‘cause I was having to put it…I couldn't even fit it in my car ‘cause I've got a Fiesta, so I was having to get my husband to take it and drop it and then pick it up and stuff like that. But then I think in a way for me again having the storage here for the first few weeks, it meant that I didn't have to take it home straightaway*. Foundation Participant 5, Female, Edinburgh

One benefit of having their bikes on site was that participants were able to access them between sessions to build a social cycling network (e.g., through additional lunchtime group rides). This led to additional demands on workplace storage, which were successfully accommodated at London and Southampton. However, session observations revealed that limited storage facilities at Edinburgh meant that participants' bikes were stored in a locked room, which was initially only accessible to Cycle Champions. Participants were therefore unable to use their bikes for group rides between sessions.

#### Acceptability

All Cycle Champions felt the delivery manual and 2-day training course gave them confidence to deliver the CNP intervention (which for many of them was a new experience):

I've never delivered training like that before. But actually, it felt quite natural, the way it happened, and everyone was really nice and listened to what you were saying. It was good. Cycle Champion 3, Male, London

The cycle champions valued the group delivery format for creating a facilitative environment where participants felt comfortable and able to support each other in gaining cycling skills and confidence:

The most effective component *was definitely the group exercises and having people of equal ability or slightly different ability but encouraging each other. As a group it worked quite well with the dynamics where there was lots of discussion between the participants, and they were encouraging each other to do things or to take part*. Cycle Champion 3, Male, London

The participants were also extremely positive about most aspects of the CNP intervention. Of the 32 participants from London and Edinburgh who completed follow-up questions about the acceptability of the CNP pilot intervention components and overall, 87.5% reported increased confidence in cycling at the end of the program and over 80% said they enjoyed it (84.4% Agree/Strongly Agree). Almost all felt the sessions were well delivered (96.9%) and the information in the handbook was clear (93.8%)—although only 53.1% reported using the handbook regularly. The opportunity to loan a bike (95.8%) [and the range of loan bikes available [87.5%]] was appreciated by those who did so (*n* = 24 respondents), with the majority (75.0%) indicating the 12-week loan period was sufficient. However, for those opting to use their own bikes (*n* = 8 respondents), 37.7% were not entirely satisfied with the quality of servicing; this largely reflects the fact that participants in London did not get their bike serviced due to issues with the local bike provider. Most participants felt the duration of the sessions (90.6%) and overall length of the program (84.4%) were appropriate and liked the balance between discussion and riding time (81.3%). Many activities within the sessions were also rated highly, including those associated with bike maintenance (71.9–87.5% Agree/Strongly Agree) and the practical components (75.0–90.6%). However, discussion of behavior change techniques—goal setting (64.5%), involving others (65.6%), and relapse prevention (56.3%)—was generally less popular. Further details of participants' responses are provided in Supplementary Table 6.

Some focus group participants further described how the CNP intervention had helped them rediscover the enjoyment of cycling, and how the skills and knowledge acquired during the initial off-road sessions allowed them to feel comfortable when moving to on-road cycling, both with the group and independently:

*I surprised myself with how confident I felt on the roads when we started to go on the roads as well because I just was really scared about that. But, no, that's been great*. Foundation Participant 6, Female, Edinburgh

Importantly, despite the Foundation and Intermediate groups in London and Edinburgh being merged from Week 5 onwards, this woman did not feel the wider range of abilities undermined the supportive culture that had been established in the early weeks:

*I think even on the group rides and stuff like that, although there was a lot of different abilities, and I would say I would probably be maybe the least…you know, like, had the least ability, but it* [merging the groups] *worked out fine. Everybody, kind of, sort of, checked in on everyone else and…you know, there wasn't really that, kind of, you know, you can't do this or we won't be able to do this on this route or whatever, kind of thing*. Foundation Participant 6, Female, Edinburgh

Participants also appreciated the fact that staff from within their office were trained as Cycle Champions to deliver the program. As one man reflected, this provided him with inspiration and motivation to cycle more:

*I think a lot of it for me was getting a, quote unquote, cyclist expertise on it […] for me it was good to see an actual person who does it semi-seriously and does it on a very, very regular basis to see their actual official way of doing things and to instil some procedure. You could tell that they were there, they wanted to share their knowledge, they were keen to share it and to instil that same passion that they had with other people*. Intermediate Participant 2, Male, Edinburgh

#### Potential effectiveness

The evidence presented above suggests the CNP pilot intervention was feasible and acceptable to Cycle Champions and participants. As Table 6 shows, the intervention also succeeded in helping participants increase their cycling by 3.0 rides (*p* < 0.001) and 43.1 min per week (*p* = 0.02), with more participants reporting increases in leisure (57.1%) and utility (40.0%) cycling, than in commuting (31.4%). Table 7 further demonstrates improvement in perceptions of cycling and walking safety, and increases in levels of vitality. Finally, internally regulated types of motivation, including identified, integrated and intrinsic motivation, all increased during the intervention.

**Table 6:** Post-intervention changes in cycling and motorized transport (Edinburgh and London sites, all *n* = 35).

	**Pre-intervention** ** (mean ±SD)**	**Post-intervention** ** (mean ±SD)**	**Change** ** (mean ±SD)**	***p*-value**
Total cycling (rides/week)	1.2 ± 2.5	4.2 ± 4.1	3.0 ± 4.6	**< 0.001**
(min/week)	12.7 ± 31.4	55.9 ± 92.6	43.1 ± 100.9	**0.02**
Utility cycling* (days/week)	0.4 ± 0.9	1.1 ± 1.4	0.8 ± 1.6	**< 0.001**
Commuting cycling (rides**/week)	0.6 ± 1.4	1.7 ± 2.7	1.1 ± 3.0	**0.04**
Leisure cycling (rides/week)	0.2 ± 0.5	1.4 ± 1.5	1.2 ± 1.6	**< 0.001**
Motorized transport use (min/week)	405 ± 464	255 ± 180	150 ± 442	**0.05**
**Participants reporting increased rides per week for different types of cycling, and overall, post-intervention (** ***n*** **, %)**				
Overall	22 (62.9)			
Utility*	14 (40.0)			
Commuting	11 (31.4)			
Leisure	20 (57.1)			

^*^Utility cycling includes shopping, running errands, school run, etc.

^**^Commuting rides are defined as one way of a return journey.

Bold values represent statistical significance (*p* < 0.05).

**Table 7:** Post intervention changes in perceptions of the environment for safe cycling and walking, wellbeing, self-esteem, vitality, and motivation (Edinburgh and London sites).

	**Participants (*n*)**	**Pre-intervention (mean ±SD)**	**Post-intervention (mean ±SD)**	**Change** ** (mean ±SD)**	***p*-value**
**Perceptions of the safety of cycling and walking**	35	43.9 ± 7.1	47.7 ± 6.3	3.8 ± 6.2	**< 0.001**
**Vitality**	34*	17.6 ± 4.8	19.4 ± 5.3	1.8 ± 5.1	**0.05**
**Self-esteem**	34*	26.3 ± 2.2	26.4 ± 1.5	0.1 ± 2.6	0.74
**Wellbeing**	34*	50.0 ± 6.6	50.7 ± 7.8	0.7 ± 5.9	0.49
**Motivation**					
Amotivation	34*	1.30 ± 0.41	1.27 ± 0.46	−0.03 ± 0.47	0.72
External regulation	34*	1.15 ± 0.30	1.25 ± 0.51	0.11 ± 0.42	0.14
Introjected regulation	34*	1.74 ± 0.91	2.49 ± 0.87	0.75 ± 1.03	**< 0.001**
Identified regulation	34*	3.44 ± 0.97	3.82 ± 0.80	0.38 ± 0.88	**0.02**
Integrated regulation	34*	1.68 ± 0.84	2.32 ± 1.12	0.65 ± 1.01	**< 0.001**
Intrinsic motivation	34*	3.50 ± 1.08	4.18 ± 0.77	0.68 ± 1.11	**< 0.001**

^*^One incomplete questionnaire.

Perceptions of the environment for safe walking and cycling, range 13–65; Wellbeing: Warwick-Edinburgh Mental Wellbeing Scale, range 14–70; Self-esteem: Rosenberg Scale, ranging 10–40; Vitality: Subjective Vitality Scale, range 4–28; Motivation: Behavioral Regulation in Exercise Questionnaire, each domain range 1–5.

Bold categories represent outcomes from the different questionnaires stated in the legend.

#### Intervention optimization

Session observations revealed that the intervention was well-delivered overall, but that some activities needed streamlined to allow the content to be delivered within the time allocated to each session. However, one recurring issue observed across multiple sessions was poor delivery of the goal setting (SMART Target) activity:

*More emphasis on SMART targets/practical targets needed. Did they achieve their individual targets etc. If they are not discussed, then participants may not feel the need to do them regularly*. Observation Foundation Session 5, London

The Cycle Champions themselves admitted to being less comfortable about delivering the behavior change components than the practical cycling components. Some suggested that the format of the goal setting activity, where participants were asked to write their personal SMART goals in their handbook each week, was a barrier to its delivery:

…*the SMART objectives that we set individuals every week, they were very difficult to police and manage. Again, because there wasn't any evidence of people bringing their handbooks week on week and we weren't obviously checking that what they said they were doing, they were doing*. Cycle Champion 1, Male, London

Despite participants being reminded to bring their handbooks to each session, it was soon evident that this was impractical:

…* there is no way in the dark in the night and outside on our bikes, you know, some people just turning up in their coats and their jackets, just turning up, there is nowhere for them to keep their book*. Cycle Champion 2, Male, Edinburgh

Therefore, following discussion with the Cycle Champions at Edinburgh, goal setting was adapted during later sessions to become a verbal activity, with a dedicated log provided to allow the Champions to record participants' individual cycling goals each week.

It had originally been envisaged that the SMART goal setting and self-monitoring of cycling would be also be supported by the CNP app, but delays in development of the self-monitoring component meant the app only became available for the Southampton delivery. However, Southampton participants remained unclear about the app's role in the intervention, with some appearing to think that it was simply to help them with route planning:

*The reason I haven't used it [the app] was because I was in the longer [Foundation] group… and it was only two weeks ago that we were all going to be going out on the road and therefore using this [the app] to ride…* (Interviewer: Did you have a look at any of the other functionality on it?) No. Female Participant 3, Foundation, Southampton

Taken as a whole, therefore, the feasibility study suggests that the CNP pilot intervention was well-implemented in the HSBC UK setting and that only a few minor changes were needed to optimize its delivery. These included further development of a simplified goal setting activity and, in order to improve practicality of delivery, combining different abilities to offer a single 9-week intervention to all participants, as shown in Table 8.

**Table 8:** Final CNP intervention: Summary of key activities for each weekly session.

**Week**	**Discussion and behavior change techniques**	**Bike maintenance**	**Practical**
1	What to wear and carry when cycling Reasons for cycling more Introduction to cycling goals In your own time activities (M-check) Introduction to cycle-to-work scheme WhatsApp social support	Introduction to the M-check	Fitting your helmet Adjusting saddle height Foot position Off-road: Starting and stopping effectively (Inc. get-ready position and braking), Slow bike race
2	Goal and activity review Introduction to SMART Targets for cycling Introduction to self-monitoring for cycling In your own time activities (dropped chain)	M-check second arm Fixing a dropped chain	Using gears Off-road: Multiple gear race
3	Cycling SMART Target review and setting Introduction to barriers In your own time activities (locking bike)	M-check final arms	Locking your bike Off-road: Cornering, Slalom game and team relay race
4	Cycling SMART Target review and setting, and activity In your own time activities (front wheel lift) review	Pumping up tire	Off-road: Avoiding obstacles, Front wheel lift, Cycling skills game
5	Cycling SMART Target review and setting Your rights on the road Involving others In your own time activities (road positioning) Reminder of cycle-to-work scheme		Off-road: Signaling, Emergency stops On-road: Road positioning, Quiet junctions
6	Cycling SMART Target review and setting Barriers and problem solving In your own time activities (traffic lights)	Chain lubrication	On-road: Traffic lights, Filtering, Roundabouts
7	Cycling SMART Target review and setting In your own time activities (changing inner tube)	Changing an inner tube (including on the rear wheel)	
8	Cycling SMART Target review and setting Overcoming setbacks In your own time activities (planning a route)		Route planning using Google Maps On-road: Group ride following planned route
9	Cycling SMART Target review Overcoming future barriers Ongoing SMART Targets Ongoing social support Graduation certificates		On-road: Group ride

## Discussion

Increasing participation in cycling requires addressing multiple barriers together (24). The CNP intervention was therefore conceived as an integrative program addressing different barriers to cycling for people who cycle infrequently. Combining multidisciplinary academic expertise with practical and contextual experience from British Cycling and HSBC UK stakeholders (including as members of the research team), as well as the target end-users, allowed us to co-develop a workplace-based intervention that was appropriate for delivery in the study setting (bank offices), succeeded in recruiting local delivery facilitators (Cycle Champions) and participants, was well-received and increased cycling participation in the short-term.

Our key innovation was to use an evidence-based approach guided by the rigorous step-by-step 6SQuID intervention development framework (42), theoretical accounts of behavior change [the Behavior Change Wheel (44) and Self-Determination Theory (45)] and evidence-based behavior change techniques (46), and move beyond single-component workplace cycling interventions (36, 39, 40, 53) to design a multi-component individual-/social-level intervention tailored to address the specific barriers to cycling for our target population (employees of a multi-national bank). These barriers were broadly similar to those observed in previous research, and included lack of cycling skills and confidence (27, 28), lack of safety (31) and social support (33), perceptions of effort (30), and cost (32). Importantly, the CNP pilot intervention attracted people from minority ethnic groups and almost as many women as men, indicating its widespread appeal. This universality means the CNP intervention is likely to be transferrable to other large employers, as well as potentially to other types of organizations, such as local authorities and community groups.

Relatively few workplace-based studies have evaluated the impact of cycling interventions on cycling behavior. Examples include an education-based intervention to increase active commuting in three large workplaces in the UK, which was effective at increasing walking but not cycling (54), and a social and individualized marketing campaign in 68 health service employees in Australia, which reported a non-significant increase (37–45%) in the proportion of staff using active commuting at 12 months (55). The mean increase in total cycling in the current study of 3.0 rides or 43.1 min per week compares favorably with these studies. Importantly, involvement in the CNP pilot intervention, as well as increasing perceptions of safety, also increased feelings of vitality, confidence and the more internalized forms of regulation that are associated with sustained behavior change (45).

Our findings also compare well with community-based cycling interventions. For example, a cycling proficiency program for adults in Australia resulted in a non-significant 10 min per week increase in cycling 2 months after the course (28); an adult cycle training program in the UK increased the proportion of participants cycling at least once a week from 40 to 61% at 3-month follow-up (56); and a 12-week group-based cycling intervention for lower-income adults in Milwaukee, USA, which included on-road education and group rides, cycle safety information and provision of bikes for participants, significantly increased the proportion of participants reporting utility cycling (by 7.2%) and cycling for fun (by 42.9%) at least twice per week from baseline to post-intervention (57). It is of note that the most successful of these previous studies (57), like CNP, also used a multi-component intervention approach.

Although regular cycling to replace other forms of transport is likely to be cost-saving (58, 59), the initial outlay of several hundred pounds to try out an activity that people are not sure is “for them” may be daunting. Therefore, the 12-week loan of a bike to support participation in CNP and transition to bike ownership was a central feature of the pilot intervention that most participants took advantage of, including some who already had their own bikes. However, although we envisaged participants would keep their loan bikes at home and bring them to work for CNP sessions (thus promoting cycling commuting), many chose to leave their loan bikes at work. As a result, although the feasibility study did demonstrate a significant increase in cycling commuting, the contribution of commuting to the total increase in cycling was less than utility and leisure cycling. In future, therefore replacing the relatively costly individual loan bikes with a fleet of shared workplace bikes (with priority given to CNP participants during the intervention) could be considered as a more cost-efficient, sustainable way of promoting a positive workplace cycling culture. Such an arrangement would also alleviate any additional pressure on workplace bike storage (as observed in Edinburgh) due to participants leaving loan bikes at work during the intervention.

Other issues identified during the feasibility study included some sessions being impacted by bad weather and poor light at Edinburgh, due to the fact that the program delivery began in autumn. At Southampton (where the program began in early February) sessions were run at lunchtime rather than in the evening. However, whilst this overcame the problem of poor light, daytime sessions and cycling practice may still be impacted by bad weather at this time of year. Therefore, Spring to early Autumn is likely to be the optimal period for intervention delivery.

In addition, although the Champions were confident about delivering the practical components of the CNP intervention, they were less comfortable about delivering the behavior change techniques, such as SMART goal setting. The fact that participants gave low ratings to many of the behavior change components may also reflect the sub-optimal delivery of these activities. Furthermore, although the CNP app was designed to help participants monitor their cycling, the app was not available for testing until the Southampton delivery. As interviews suggested participants did not use the app for its intended purpose, it will not be used in the optimized version of the program. Further refinement (including some co-development with Cycle Champions and users) may therefore be required to optimize engagement with the behavior change techniques during the CNP intervention.

Finally, the additional complexity and Cycle Champion commitment required to deliver two versions of the program meant that Foundation and Intermediate group sessions were often merged. Therefore, despite the demand for the two different levels in the program development workshops, a more streamlined single-level, 9-week foundation-level program appears to be more feasible to deliver in practice.

This study had a number of strengths. Most importantly, co-development of the intervention with stakeholders, including staff and managers from across HSBC UK, and inclusion of bank and British Cycling representatives on the research team ensured the pilot intervention could be delivered within the practical opportunities and constraints of the organizational context. In addition, selection of different offices to represent geographical diversity across the UK, supports the wider implementation of the CNP intervention necessary for a future full-scale randomized controlled trial and post-research roll out. Finally, in two of the three feasibility offices, women staff members volunteered to be Cycle Champions. Nevertheless, more could be done to promote the Cycle Champion role to experienced women cyclists to provide a range of cycling role models within each office whose lifestyles and personal commitments are relatable to all participants. The main limitations were lack of a control group and the suspension of the study in March 2020 due to COVID-19, meaning that no participants from the third delivery site contributed to post-program data collection, and that we were unable to conduct longer-term follow-up. Nevertheless, the fact that the forms of motivational regulation associated with behavior change maintenance increased significantly is promising in relation to the intervention's potential to support sustained behavior change.

## Conclusion

The CNP intervention was co-developed iteratively with key stakeholders and end-users drawing on experiential and theoretical evidence. This resulted in an intervention that was successful (at least in the short term) in increasing cycling and improving perceptions of safety, vitality, confidence and motivation to cycle. In addition, the intervention was feasible when delivered to staff within the offices of a UK-based multinational bank, and acceptable to both women and men, and across different ethnicities. Given that the barriers to cycling highlighted by bank employees were similar to those identified in the general population, the CNP intervention has potential to be delivered through different organizations, including other large employers and local authorities as part of their wellbeing and active travel initiatives. The long-term effectiveness and cost-effectiveness of this approach should be tested in a full-scale randomized controlled trial.

## Data availability statement

The raw data supporting the conclusions of this article will be made available by the authors, without undue reservation.

## Ethics statement

The studies involving human participants were reviewed and approved by University of Glasgow College of Social Sciences Ethics Committee (Ref. 400170195) and University of Glasgow College of Medical, Veterinary and Life Sciences Ethics Committee (Ref. 200180138). The patients/participants provided their written informed consent to participate in this study.

## Author contributions

CG, JG, PK, GB, and EM contributed to conception and design of the study. GL, CG, HC, and JG designed the intervention delivery materials, were involved in data collection, and wrote the first draft of the manuscript. GL and CG supported the Cycle Champion training. GL, HC, JG, CB, and CG undertook data analysis. All authors contributed to intervention development, contributed to manuscript revision, read, and approved the submitted version.

## Funding

This work was supported by a Research Grant from British Cycling and HSBC UK.

## Conflict of interest

The nature of the co-design of the Cycle Nation Project intervention meant that the funders, British Cycling and HSBC UK, were intimately involved in the design and delivery of the intervention, and representatives from both organizations are authors on the paper. All data analysis was performed by researchers at the University of Glasgow without input from British Cycling or HSBC UK. Authors SB and JP were employed by British Cycling, and authors SR and LH are employees of HSBC UK. The remaining authors declare that the research was conducted in the absence of any commercial or financial relationships that could be construed as a potential conflict of interest.

## Publisher's note

All claims expressed in this article are solely those of the authors and do not necessarily represent those of their affiliated organizations, or those of the publisher, the editors and the reviewers. Any product that may be evaluated in this article, or claim that may be made by its manufacturer, is not guaranteed or endorsed by the publisher.
