# Supplementary material for: Development and optimisation of a multi-component workplace intervention to increase cycling for the Cycle Nation Project
Source: Front Sports Act Living. 2022 Oct 26;4:857554. doi: 10.3389/fspor.2022.857554 (PMC9643150; doi:10.3389/fspor.2022.857554)
Supplement: Supplementary file 1 [file Data_Sheet_1.docx]

**Supplementary Material**

Supplementary Table 1. Theoretical mapping of candidate CNP intervention components against modifiable factors

| **Intervention function** | **Action  category** | **Candidate CNP intervention components (Action type/Facilitator)** | **Modifiable factor(s)** | **Final CNP component (Y/N)** |
| --- | --- | --- | --- | --- |
| **EDUCATION** Increasing knowledge or understanding | **Increasing knowledge or understanding of benefits of cycling** | | | |
|  |  | *Information on time-saving benefits of cycling (SR)* | Lack of experience | Y |
|  |  | *Information on cost benefits of cycling (SR)* | Cost of bike + Cost of equipment | Y |
|  |  | *Information on time taken to cycle route (e.g., in app) (FG)* | Lack of experience + Unsure how to plan a route | Y |
|  | **Increasing knowledge or understanding of cycling safety** | | | |
|  |  | *Information on cycling safely (SR, FG)* | Unsure how to cycle on road + Lack of experience + Lack of confidence | Y |
|  |  | *Information on cycle etiquette (FG)* | Lack of experience + Lack of confidence | Y |
|  |  | *Acting out travel scenarios (SR)* | Unsure how to cycle on road + Lack of experience | Y |
|  | **Route planning/personal and individualised travel planning** | | | |
|  |  | *Information on accessibility and local routes (SR,FG)* | Unsure how to plan a route | Y |
|  |  | *Travel and safe-route maps (SR,FG)* | Unsure how to plan a route | Y |
|  |  | *Digital cycling apps (SR,FG)* | Unsure how to plan a route | Y |
|  |  | *Cycling website (SR,FG)* | Unsure how to plan a route | Y |
|  | **Practical or instrumental information** | | | |
|  |  | *Local showering options (SR,FG)* | Lack of showers | Advice* |
|  |  | *Local storage options (SR,FG)* | Lack of secure storage | Advice* |
|  |  | *General practical ‘Everything you need to know about cycling’ information (SR)* | Lack of experience | Y |
|  | **Signposting to cycling resources/organisations** | | | |
|  |  | *Cycling-related contacts (SR)* | Lack of experience | Y |
|  |  | *Information on local cycle clubs/groups (e.g., for different abilities) (FG)* | Others not interested in cycling + No cycling role models/mentors | N |
| **PERSUASION** Communication to induce positive/ negative feelings or stimulate action | **One to one counselling** | | | |
|  |  | *Barrier identification and problem solving (SR)* | All individual/social factors | N |
|  | **Group counselling** | | | |
|  |  | *Group counselling (including barrier identification and problem solving) to increase cycling (SR)* | All individual/social factors | Y |
|  | **Travel diaries** | | | |
|  |  | *Feedback on individualised travel diaries (SR)* | Lack of confidence + Unsure how to cycle on road | N |
| **INCENTIVISATION** Creating expectation of award | **Material** | | | |
|  |  | *Bikes for attending sessions (SR)* | Cost of bike | Y |
|  | **Financial** | | | |
|  |  | *Subsidy, salary sacrifice, tax free loan for buying bike and equipment (SR,FG)* | Cost of bike + Cost of equipment | Y** |
|  |  | *Retail and repair discounts (SR,FG)* | Cost of bike + Cost of equipment | N |
|  |  | *Cash incentive for cycling (e.g., equal to cost of parking) (SR,FG)* | Cost of bike + Cost of equipment | N |
|  |  | *Cycling-related gifts* | Cost of equipment | Y |
|  |  | *Free bike service for taking part* | Cost of bike + Lack of bike maintenance skills | Y |
|  |  | *Bike vouchers (SR,FG)* | Cost of bike | N |
|  | **Gamification/challenges** | | | |
|  |  | *Goal setting and personal challenges (SR)* | Unsure how to cycle on road + Unsure how to plan a route + Lack of bike maintenance skills + Lack of experience + Lack of confidence + Hills + Distance | Y |
|  |  | *Reward schemes for cycling (SR,FG)* | Lack of experience | N |
|  |  | *Awards and certificates (SR)* | Lack of experience | Y |
|  |  | *Leader boards and prizes (SR)* | Lack of experience + Lack of confidence | N |
|  |  | *Within/between workplace challenge (SR/FG)* | Lack of experience | N |
|  |  | *Active games (SR)* | Lack of experience + Lack of confidence | Y |
| **TRAINING** Imparting skills | **Training courses and sessions** | | | |
|  |  | *Cycle skills, proficiency and safety training and courses (SR,FG)* | Lack of experience + Unsure how to cycle on road + Lack of confidence | Y |
|  |  | *e-Bike skills and proficiency training and courses (SR,FG)* | Lack of experience + Lack of confidence + Hills + Distance | Y |
|  |  | *Maintenance skills courses (FG)* | Lack of bike maintenance skills | Y |
|  |  | *Independent skills practice (SR)* | Lack of experience + Unsure how to cycle on road + Lack of confidence | Y |
| **ENVRONMENTAL RESTRUCTURING** 1) Changing the physical environment | **Bicycle storage** | | | |
|  |  | *Provision of cycle storage facilities (SR,FG)* | Lack of secure storage | N |
|  | **Bicycle maintenance facilities** | | | |
|  |  | *Tools, workshop area, stands for bicycle repair (SR,FG)* | No access to bike spares/tools | N |
|  |  | *Vending machines for bicycle repair supplies (SR)* | No access to bike spares/tools | N |
|  | **Facilities for cyclist** | | | |
|  |  | *Changing facilities (e.g., showers, lockers) (SR,FG)* | Lack of showers + Lack of lockers | N |
|  | **Safety/security** | | | |
|  |  | *Security cameras at bike parking (SR)* | Lack of secure storage | N |
| **ENVRONMENTAL RESTRUCTURING**  2) Changing the social environment | **Personnel** | | | |
|  |  | *Bike maintenance technician to visit workplace (FG)* | No access to bike spares/tools + Lack of bike maintenance skills | N |
|  |  | *Employment of external cycling instructor (SR)* | No cycling role models/mentors | N |
|  | **Large events and mass participation** | | | |
|  |  | *Bike to work days (SR)* | Others not interested in cycling + No cycling role models/mentors | N |
|  |  | *Large, organised ride events (SR,FG)* | No cycling role models/mentors + Others not interested in cycling | N |
|  | **Group cycling** | | | |
|  |  | *Led group bike rides (SR,FG)* | No cycling role models/mentors + Others not interested in cycling | Y |
|  |  | *"Bike buses" (organised group travel to/from work)* | No cycling role models/mentors + Others not interested in cycling | N |
|  | **Workplace or organisational policies** | | | |
|  |  | *General workplace or organisational policies (SR,FG)* | Professional dress code | N |
|  |  | *Workplace travel plans (SR)* | Unsure how to plan a route | N |
|  |  | *Organisation task force on cycling (SR, FG)* | Professional dress code + No access to bike spares/tools | N |
|  |  | *Training internal staff to become certified cycling instructor (SR)* | No cycling role models/mentors | Y |
|  |  | *Relax rules/attitudes towards cycle clothes (FG)* | Professional dress code | N |
|  |  | *Staff can finish work early to avoid traffic (FG)* | Lack of confidence | N |
| **MODELLING** Providing an example for people to aspire to or imitate | **"Buddying" systems** | | | |
|  |  | *Bike mentoring - pairing less experienced cyclist with more experienced cyclist (SR, FG)* | No cycling role models/mentors | N |
|  |  | *Online eBuddy system (SR, FG)* | No cycling role models/mentors | N |
| **ENABLEMENT** Increasing means/reducing barriers to increase capability or opportunity | **Provision of bike accessories** | | | |
|  |  | *Safety equipment (helmets, lights, reflective strips) (SR,FG)* | Cost of equipment | Y |
|  |  | *Cycling related merchandise (SR)* | Cost of equipment | N |
|  | **Provision of eBikes** | | | |
|  |  | *Loan of eBike to use during intervention/eBike trial before purchase (SR,FG)* | Cost of bike + Distance + Hills | Y |
|  |  | *Workplace 'errand' eBikes (SR)* | Cost of bike + Lack of secure storage + Distance + Hills | N |
|  | **Provision of bikes** | | | |
|  |  | *Provision of bike to keep (SR,FG)* | Cost of bike | N |
|  |  | *Short term hire or lease of bike during intervention (SR,FG)* | Cost of bike | Y |
|  |  | *Earning a bicycle to keep following refurbishment/recycling (SR)* | Cost of bike | N |
|  |  | *Provision of shared bikes (FG)* | Cost of bike | N |
|  |  | *Bike donation targeting lower socioeconomic groups (SR)* | Cost of bike | N |
|  | **Cycle share schemes** | | | |
|  |  | *Workplace 'errand' bikes (SR,FG)* | Cost of bike + Lack of secure storage | N |
|  |  | *Provision of shared bikes* | Cost of bike + Lack of secure storage | N |
|  |  | *Information on how to access shared cycle schemes (FG)* | Cost of bike + Lack of secure storage + Lack of knowledge of cycle share schemes | Y |
|  | **Provision of bike maintenance** | | | |
|  |  | *General bike maintenance (SR,FG)* | Lack of bike maintenance skills | Y |
|  |  | *Bike repairs (SR,FG)* | Cost of bike + Lack of bike maintenance skills | Y |

SR=Scoping Review; FG=Focus Group. *Findings reported back to HSBC UK as potential organisational level intervention. **Existing HSBC UK cycle-to-work scheme.

Supplementary Table 2. Sessions observed by members of the CNP research team

|  | **Sessions observed** | | | | | | | | |
| --- | --- | --- | --- | --- | --- | --- | --- | --- | --- |
|  | **1** | **2** | **3** | **4** | **5** | **6** | **7** | **8** | **9** |
| **London** |  |  |  |  |  |  |  |  |  |
| Foundation | x | x | x | x | x | x | x | x* | x |
| Intermediate | - | - | - | x | ** | ** | ** | ** | ** |
| **Edinburgh** |  |  |  |  |  |  |  |  |  |
| Foundation | x | x | x | x | x | - | x | - | x |
| Intermediate | - | - | - | x | - | x | - | x | ** |
| **Southampton** |  |  |  |  |  |  |  |  |  |
| Foundation | x | x | x | - | x | n/a | n/a | n/a | n/a |
| Intermediate | - | - | - | x | - | n/a | n/a | n/a | n/a |
| * Including attendance at extra session run indoors to accommodate adverse weather; **Foundation  and intermediate sessions merged; n/a: sessions not delivered due to COVID-19 | | | | | | | | | |

Supplementary Table 3. Description of themes emerging from the participant focus groups and Cycle Champion interviews and participant exit interviews

| **Theme** | **Research question** | **Description** |
| --- | --- | --- |
| **Recruitment** | Feasibility | Reasons for joining, personal goals |
| **Adherence** | Feasibility | Commitment every week, feelings of guilt, reasons for non-attendance |
| **Practical aspects** | Feasibility | Practicality of doing intervention, views of space, locations, bikes, timing, storage |
| **Acceptability** | Acceptability and optimisation | Likes/dislikes, what worked well/did not work well |
| **Impact** | Potential effectiveness | Anything said about changed cycling, wellbeing, motivation, self-esteem, feelings of vitality |
| **Post-programme maintenance** | Potential effectiveness | Anything said about ongoing impact/intentions (including purchasing a bike) |

Supplementary Table 4: Session attendance at each of the CNP feasibility offices

|  | **Session attendance [n (%)]** | | | | | | | | | **Sessions attended**  **[n attended/**  **total n available (%)]** | **Completion rates****  **Foundation + Intermediate combined (n(%))** |
| --- | --- | --- | --- | --- | --- | --- | --- | --- | --- | --- | --- |
|  | **1** | **2** | **3** | **4** | **5** | **6** | **7** | **8*** | **9** |  |  |
| **London** |  |  |  |  |  |  |  |  |  |  |  |
| Foundation (n=9) | 9  (100.0) | 8  (88.8) | 7  (77.7) | 6  (66.6) | 3  (33.3) | 3  (33.3) | 6  (66.6) | 6  (66.6) | 2  (22.2) | 51/81  (63.0) | 10  (71.4) |
| Intermediate (n=5) | - | - | - | 3  (60.0) | 3  (60.0) | 2  (40.0) | 4  (80.0) | 3  (60.0) | 3  (60.0) | 18/30  (60.0) |  |
| **Edinburgh** |  |  |  |  |  |  |  |  |  |  |  |
| Foundation (n=14) | 13  (92.8) | 10  (71.4) | 11  (78.6) | 9  (64.3) | 8  (57.1) | 10  (71.4) | 12  (85.7) | 10  (71.4) | 9  (64.3) | 92/126  (73.0) | 17  (54.8) |
| Intermediate (n=13) | - | - | - | 5  (38.5) | 6  (46.2) | 6  (46.2) | 7  (53.8) | 10  (76.9) | 8  (61.5) | 42/78  (53.8) |  |
| **Southampton** |  |  |  |  |  |  |  |  |  |  |  |
| Foundation (n=16) | 16 (100.0) | 15  (93.8) | 15  (93.8) | 16  (100.0) | 15  (93.8) | - | - | - | - | 77/80  (96.2) | NA |
| Intermediate (n=7) | - | - | - | 7  (100.0) | 7  (100.0) | - | - | - | - | 14/14  (100.0) |  |
| *Session 8 in London was delivered over two weeks due to bad weather (Week 1, education session delivered indoors; Week 2, on road practical ride)  **Completion was defined as attendance at two thirds of sessions | | | | | | | | | | | |

Supplementary Table 5. Types of bikes loaned at each CNP feasibility offices and overall

|  | **London (n, %)** | **Edinburgh (n, %)** | **Southampton (n, %)** | **Total (n, %)** |
| --- | --- | --- | --- | --- |
| e-bike | 3 (30.0) | 5 (20.0) | 2 (9.5) | 10 (17.9) |
| Hybrid | 2 (20.0) | 16 (64.0) | 12 (57.1) | 30 (53.6) |
| Road bike | 3 (30.0) | 4 (16.0) | 4 (19.0) | 11 (19.6) |
| Folding bike | 2 (20.0) | 0 (0.0) | 3 (14.3) | 4 (8.9) |
| **Total bikes loaned** | 10 | 25 | 21 | 56 |

Supplementary Table 6. Participant views of the CNP Intervention (n = 32)

|  | **Disagree/Strongly Disagree (%)** | **Neutral**  **(%)** | **Agree/Strongly Agree (%)** |
| --- | --- | --- | --- |
| **Overall views** | | | |
| I enjoyed taking part in the programme | 9.4 | 6.3 | 84.4 |
| I thought 9 weeks (or 6 weeks for the intermediate course) was an appropriate length for the course | 6.3 | 9.4 | 84.4 |
| I thought that 90 minutes was an appropriate length for each session | 3.1 | 6.3 | 90.6 |
| I felt the balance between discussion and practical riding time was good | 12.5 | 6.3 | 81.3 |
| The course handbook was clear and easy to follow | 0.0 | 6.3 | 93.8 |
| I used the course handbook regularly throughout the programme | 18.8 | 28.1 | 53.1 |
| I found the content of the course appropriate and relevant | 0.0 | 3.1 | 96.9 |
| I thought the cycle champion delivered the course well | 0.0 | 3.1 | 96.9 |
| I felt supported by the cycle champions at my office | 12.5 | 18.8 | 68.8 |
| I now have more confidence to cycle my bike | 6.4 | 3.1 | 87.5 |
| **Views on specific CNP programme components** | | | |
| *Bike Maintenance* | | | |
| I found the M check useful* | 3.1 | 16.1 | 80.6 |
| I found the information on fixing a dropped chain useful | 0.0 | 12.5 | 87.5 |
| I found learning how to pump up my tyres useful | 3.1 | 25.0 | 71.9 |
| I found the puncture clinic useful | 3.1 | 18.8 | 78.1 |
| *Practical* | | | |
| I found the advice on starting and stopping useful | 3.1 | 15.6 | 81.3 |
| I found the advice on changing gears useful | 6.3 | 15.6 | 78.1 |
| I found learning how to lock my bike useful | 3.1 | 21.9 | 75.0 |
| I found the off-road cycling useful | 6.3 | 18.8 | 75.0 |
| I found the on-road cycling useful | 0.0 | 9.4 | 90.6 |
| *Discussion and behaviour change techniques* | | | |
| I found the discussion on road safety useful | 0.0 | 9.4 | 90.6 |
| I found the route planning session useful | 3.1 | 25.0 | 71.9 |
| I felt the information on accessing a cycle-share scheme was clear | 0.0 | 21.9 | 78.1 |
| I found the practical SMART targets useful (i.e. cycling each week)* | 9.7 | 25.8 | 64.5 |
| I found the theoretical SMART targets useful (e.g. practicing changing my inner tube)** | 13.3 | 23.3 | 63.3 |
| I found thinking about overcoming barriers to cycling useful | 3.1 | 15.6 | 81.3 |
| I found the discussion about ‘involving others” in cycling useful | 3.1 | 31.3 | 65.6 |
| I found the discussions on relapse prevention useful | 0 | 43.8 | 56.3 |
| *Provision of bikes and equipment* | | | |
| I liked that I could loan a bike for the duration of the programme*** | 0 | 4.2 | 95.8 |
| I felt that the bike loan was for a long enough period of time*** | 12.5 | 12.5 | 75.0 |
| The choice of loan bikes was good*** | 0 | 12.5 | 87.5 |
| I found the arrangements for getting my bike serviced straightforward**** | 0 | 25.0 | 75.0 |
| I found the quality of the servicing carried out to be good**** | 12.5 | 25.0 | 62.5 |
| I thought the advice on how I could buy a new bike through the bike to work scheme was clear | 3.1 | 9.4 | 87.5 |
| I was happy with the helmet I received | 3.1 | 34.4 | 62.5 |
| I was happy with the lock I received | 0 | 28.1 | 71.9 |
| I found the lights I received during the programme useful | 9.4 | 9.4 | 81.3 |
| I found the waterproof phone carrier I received useful | 0 | 3.1 | 96.9 |

*n=31, **n=30, *** for those who had a loaned bike (n=24), ****for those who used their own bike on the programme (n=8)
